# SOX19b regulates the premature neuronal differentiation of neural stem cells through EZH2-mediated histone methylation in neural tube development of zebrafish

**DOI:** 10.1186/s13287-019-1495-3

**Published:** 2019-12-16

**Authors:** Xian Li, Wenjuan Zhou, Xinyue Li, Ming Gao, Shufang Ji, Wenyu Tian, Guangyu Ji, Jingyi Du, Aijun Hao

**Affiliations:** 1 Key Laboratory of the Ministry of Education for Experimental Teratology, Shandong Provincial Key Laboratory of Mental Disorders, Department of Human Anatomy and Histoembryology, School of Basic Medical Sciences, Shandong University, 44#, Wenhua Xi Road, Jinan, 250012 Shandong China; 2 Foot and Ankle Surgery Center of Shandong University and Department of Hand and Foot Surgery, The Second Hospital of Shandong University, Jinan, Shandong China; 3 National Research Center for Assisted Reproductive Technology and Reproductive Genetics, Center for Reproductive Medicine, Shandong University, Jinan, China

**Keywords:** Zebrafish, Neural tube defects, Sox19b, Neural stem cells

## Abstract

**Objective:**

Neural tube defects (NTDs) are the most serious and common birth defects in the clinic. The SRY-related HMG box B1 (SoxB1) gene family has been implicated in different processes of early embryogenesis. Sox19b is a maternally expressed gene in the SoxB1 family that is found in the region of the presumptive central nervous system (CNS), but its role and mechanism in embryonic neural stem cells (NSCs) during neural tube development have not yet been explored. Considering that Sox19b is specific to bony fish, we intended to investigate the role and mechanism of Sox19b in neural tube development in zebrafish embryos.

**Material and methods:**

Morpholino (MO) antisense oligonucleotides were used to construct a Sox19b loss-of-function zebrafish model. The phenotype and the expression of related genes were analysed by in situ hybridization and immunolabelling. Epigenetic modifications were detected by western blot and chromatin immunoprecipitation.

**Results:**

In this study, we found that zebrafish embryos exhibited a reduced or even deleted forebrain phenotype after the expression of the *Sox19b* gene was inhibited. Moreover, we found for the first time that knockdown of Sox19b reduced the proliferation of NSCs; increased the transcription levels of *Ngn1*, *Ascl1*, *HuC*, *Islet1*, and cyclin-dependent kinase (CDK) inhibitors; and led to premature differentiation of NSCs. Finally, we found that knockdown of Sox19b decreased the levels of EZH2/H3K27me3 and decreased the level of H3K27me3 at the promoters of *Ngn1* and *ascl1a*.

**Conclusion:**

Together, our data demonstrate that Sox19b plays an essential role in early NSC proliferation and differentiation through EZH2-mediated histone methylation in neural tube development. This study established the role of transcription factor Sox19b and epigenetic factor EZH2 regulatory network on NSC development, which provides new clues and theoretical guidance for the clinical treatment of neural tube defects.

## Background

The embryonic neural tube is the precursor of the central nervous system. Neural tube development is a continuous development process from the appearance of the nerve plate to the closure of the nerve tube [1]. In this process, congenital neurodevelopmental diseases caused by neural tube formation and closure disorder are called neural tube defects (NTDs) [2, 3]. NTDs are the most serious and common birth defects in clinical practice and are the main causes of disability and death in newborns [4, 5]. Previous studies have shown that the pathogenesis of NTDs is mainly the result of genetic factors, environmental factors, and maternal factors. The clinical manifestations of NTDs include brain deformity, cerebral dysplasia, encephalocele, spina bifida, and spinal meningocele [6–8]. However, the molecular mechanisms of NTDs are not yet fully understood.

The proliferation, differentiation, and migration of neural stem cells (NSCs) are the cellular basis of neural tube development. An increasing number of studies have shown that exploring the regulatory mechanism of proliferation and differentiation of NSCs may be the key to revealing the pathogenesis of NTDs [9–12]. The transition of NSCs from proliferation to neuronal commitment is a multi-step process; pluripotent NSCs exit the cell cycle due to the dynamic interaction of extracellular and intracellular signals. Eventually, neurons with different cellular characteristics are produced [13, 14]. The SRY-related HMG box B1 (SoxB1) family is the most important transcription factor regulating the proliferation and differentiation of NSCs; the main SoxB1 family members are Sox1, Sox2, and Sox3 [15, 16]. Indeed, studies have shown that the SoxB1 family is closely related to NTDs. Loss or mutation of *sox2* in humans can cause hypoplasia of the hippocampus, corpus callosum, cortex, and hypothalamus; ventricular enlargement; and anophthalmia [17, 18]. A new human study also found that in 75 patients with NTD, 16% had a complete loss of sox2 [19].

Zebrafish have the advantages of in vitro development, transparent embryo, and rapid development, which makes it a good animal model for studying the development of neural tube [20, 21]. The SoxB1 family in zebrafish includes Sox1a, Sox1b, Sox2, Sox3, Sox19a, and Sox19b, where Sox19a and Sox19b are specific to bony fish [22, 23]. The expression of Sox1a and Sox1b is not observed until the caudal bud stage is mainly expressed in the lens and is not related to the development of the neural tube. In our study, we focused on the other 4 SoxB1 family members. In combination with previous reports and our results, the expression characteristics of the SoxB1 family in zebrafish are as follows: only Sox19b was highly expressed during the maternal phase, Sox3 and Sox19a were expressed initially in the 1000 cell phase, and Sox2 was expressed at the beginning of the 30% epiboly phase. All 4 were expressed until 48 hpf. Sox2, Sox3, Sox19a, and Sox19b were expressed in the neuroectodermal region until the 75% of epiboly phase, but the expression of Sox2 and Sox3 changed as development progressed. Sox2 is mainly expressed in the anterior part of the neural tube, optic vesicle, and retina, while Sox3 is mainly expressed in the posterior brain, lens, and olfactory system; only Sox19a and Sox19b are expressed specifically throughout the neural tube [23]. However, the role of Sox19b in neural tube development has not been systematically investigated. The maternal expression of Sox19b and the specific neural tube distribution, which is similar to that of Sox2 in mammals, attracted our attention.

In this study, we knocked down Sox19b in zebrafish embryos using morpholino (MO) antisense oligonucleotides and confirmed that Sox19b is functionally important in neural tube development. More importantly, we found that knockdown of Sox19b could lead to a decrease in the proliferation of NSCs in the neural tube and premature differentiation. In neural development, Sox19b is essential for the proper regulation of ngn1, ascl1, and her3. Moreover, in terms of mechanism, we found that Sox19b induces high levels of histone H3K27me3 through the activity of EZH2, maintains the levels of histone H3K27me3, promotes cell division and proliferation, and maintains the stem cell pool. In this study, the networks of transcription factors and epigenetic factors that regulate the fate of NSCs were established to deepen the understanding of NSC proliferation and differentiation. These findings indicate that Sox19b plays a central role in the development of neural tubes, and provide new ideas and clues for the clinical treatment of NTDs.

## Materials and methods

### Zebrafish maintenance

AB strains of zebrafish were maintained and bred according to standard procedures. The ESEN zebrafish culture system (ESEN EnvironScience, Beijing, China) was used to control the temperature and the day-night cycles. All embryos were fertilized and incubated at 28.5 °C without crowding (five to ten embryos per millilitre).

### Microinjection of morpholino antisense oligonucleotides

Two morpholine ring modified antisense oligonucleotides for Sox19b were designed in this study, and they targeted the 5′ untranslated region and the translation initiation region of Sox19b mRNA. The sequence is 5′-TACATCATGCCACTTCTCGCTTTGA-3′, 5′-GTCTTCAGCTCGTGCTCCATCATGC-3′. The standard control for experiments was the sequence 5′-GTGTTGAGGTCGTCCTCCATCATCC-3′. All oligonucleotides were diluted with distilled water and stored at − 20 °C. MO oligonucleotides were injected into zebrafish embryos at the one-cell stage with 5–9 ng/embryo, and the injection volume was generally 2–3 nl. The specificity and efficiency of the Sox19b MO oligonucleotides in zebrafish were determined after coinjection of a Sox19b MO or control MO with a GFP-tagged Sox19b mRNA (Fig. 2a). Zebrafish full-length Sox19b cDNA was amplified using 5′-GCGGATCCATGATGTACAGCATGATGGA-3′ forward and 5′-GCTCTAGAGATGTGAGTGAGGGGAACAGTT-3′ reverse primers, and the cDNA was cloned into pCS2-GFP expression vectors using BamHI and XbaI restriction sites.

### Reverse transcription polymerase chain reaction and quantitative real-time PCR

**Fig. 1:**
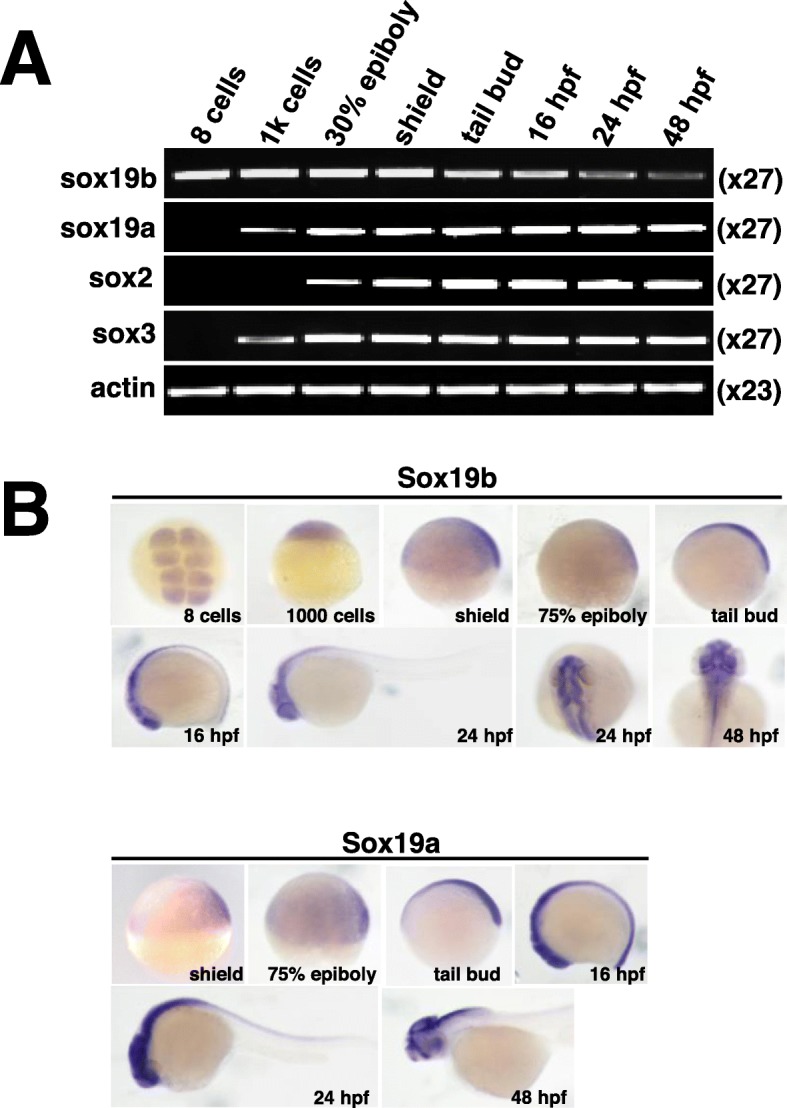
Expression of the SoxB1 family of genes during zebrafish embryonic development. **a** RT-PCR was used to analyse the expression level of the *SoxB1* family of genes during zebrafish developmental stages, ranging from 8 cells to 48 hpf. **b** Expression analysis of *Sox19b* and *Sox19a* in zebrafish was determined by whole-mount in situ hybridization at the indicated stages (from 8 cells to 48 hpf)

Total RNA was extracted using an RNeasy kit (Qiagen, USA) according to the manufacturer’s instructions. Total RNA was treated with DNase I. The cDNA was synthesized with a RevertAid™ First Strand cDNA Synthesis Kit (Thermo Fisher Scientific) following the manufacturer’s protocol. PCR was performed with EasyTaq PCR SuperMix (TransGen, Beijing, China). The protocol was performed as follows: initial denaturation at 95 °C for 5 min, then 95 °C for 30 s, 56 °C for 40 s, 72 °C for 30 s, repeated for 23 or 27 cycles (marked in Fig. 1a). The primer sequences were as follows: *Sox19a* (forward, 5′-catgtccatggtgaaaccag-3′; reverse, 5′-cgtaccggtgaggtaatgct-3′), *Sox19b* (forward, 5′-aaatatcctcttgcagcggg-3′; reverse, 5′-ctgttcatgtagggctgtgc-3′), *Sox2* (forward, 5′-gaaccccaaaatgcacaattcg-3′; reverse, 5′-acttgtccttcttcatcagggt-3′), and *Sox3* (forward, 5′-ccattccgcagtccaaca-3′; reverse, 5′-gattctcctgagccatcttc-3′). Real-time PCR was performed with SYBR Green Realtime PCR Master Mix (TOYOBO CO., Ltd., Japan). The primer sequences were as follows: *Ngn1* (forward, 5′-cttcgctcacaactacatctgg-3′; reverse, 5′-cactacgtcggtttgcaagtat-3′), *Huc* (forward, 5′-taacggccctgtcattagca-3′; reverse, 5′-cgtgttgatagccttgtcgg-3′), *ascl1a* (forward, 5′-cgggtgaagcttgtgaacaa-3′; reverse, 5′-ttttgggagatggtgggtga-3′), *Her3* (forward, 5′-tacacttcgacgaccacaca-3′; reverse, 5′-tgaagtgtgtggtttctgcc-3′), *actin* (forward, 5′-tcttccagccttccttcctg-3′; reverse, 5′-tggaaggagcaagagaggtg-3′), *p21* (forward, 5′-gtgtcaggaaaagcagcaga-3′; reverse, 5′-gacgcttcttggcttggtag-3′), *p27* (forward, 5′-ttcgcttgtctaatggcagc-3′; reverse, 5′-gtgcgtggaaaagtcgaagt-3′), *p57* (forward, 5′-acttttcctctcctcacccg-3′; reverse, 5′-aaatcgcctcccactcgtaa-3′), *EZH2* (forward, 5′-tgagaccaccagctcttcag-3′; reverse, 5′-ggtctttgtgccgatgagtc-3′), *fgf3* (forward, 5′-cccaagggcgccttgtgccaggg-3′; reverse, 5′-ccgtgttttaaagcccctcctgg-3′), *fgf8* (forward, 5′-ttcacggttgagttatctattcc-3′; reverse, 5′-agtcttttcccagaccatttttc-3′), and *er81* (forward, 5′-gaaaacttggggctccacgggct-3′; reverse, 5′-gggaaggggatgctgggctctga-3′). The expression of β-actin was used as an internal control, and the 2^−ΔΔCT^ method was used to calculate the changes in the gene expression levels.

### In situ hybridization and immunolabelling

Briefly, antisense RNA probes were synthesized with digoxigenin RNA labelling kits (Roche). Embryos were collected at different stages and were fixed in 4% paraformaldehyde. The fixed embryos were pre-treated with proteinase K, washed with PBST, and then the egg membrane was removed. The embryos were prehybridized in Hyb^−^ for 5 min and Hyb^+^ for 4 h at 65 °C; then, they were incubated with antisense RNA probes (1 ng/μl) overnight. The following antisense RNA probes were used: *Sox19a*, *Sox19b*, *otx2*, *pax2a*, *PCNA*, *Ngn1*, *Huc*, and *Islet1.* The next day, the embryos were washed in washing liquid I, II, and III, and blocking solution was used to block the nonspecific binding sites. Then, the embryos were incubated with anti-DIG-AP (Roche) at 4 °C overnight. On the third day, INT/BCIP was used for colour detection, and images were acquired with a stereomicroscope (Olympus SZX16).

For immunolabelling, embryos were fixed in 4% paraformaldehyde and embedded in OCT. Cryostat sections were cut at 8 μm in thickness and labelled with primary antibodies overnight at 4 °C, which was followed by incubation with appropriate secondary antibodies: horseradish peroxidase (HRP)-conjugated goat anti-rabbit IgG antibody (1:500, AQ132P; Sigma-Aldrich) or Alexa Fluor 594 goat anti-rabbit IgG secondary antibody (1:500, A-11037; Invitrogen). The primary antibodies were used as follows: rabbit anti-HuC (1:200, GTX128365; GeneTex) and rabbit anti-PHH3 (1:200, #3377; Cell Signaling Technology). Images were acquired with a fluorescence microscope (Olympus IX71) after immunostaining.

### Western blotting

Zebrafish embryos were homogenized in RIPA buffer with protease and phosphatase inhibitors (Beyotime Institute of Biotechnology, Shanghai, China). The supernatants were collected by centrifuging at 12000 rpm at 4 °C for 15 min. The supernatants were mixed with loading buffer, and equal amounts of protein were loaded onto 15% SDS-PAGE gels. When the separated proteins were transferred to the PVDF membranes, the PVDFs were incubated with primary antibodies and then with an HRP-conjugated goat anti-rabbit IgG antibody (1:5000, AQ132P; Sigma-Aldrich). The primary antibodies were used as follows: rabbit anti-H3 (1:1000, #4499; Cell Signaling Technology), rabbit anti-acetyl-H3 (1:1000, #06-599; Millipore), rabbit anti-H3K27me3 (1:1000, #9733; Cell Signaling Technology), rabbit anti-H3K9me3 (1:1000, # 13969; Cell Signaling Technology), rabbit anti-GFP (1:1000, #2956; Cell Signaling Technology), and rabbit anti-β-actin (1:2000, SAB2100037; Sigma-Aldrich). Protein bands were analysed by densitometry using Quantity One.

### Chromatin immunoprecipitation assay

Chromatin immunoprecipitation (ChIP) was conducted with an EZ-ChIP kit (Merck Millipore) according to the manufacturer’s protocol. Briefly, zebrafish embryos were fixed with 1% formaldehyde for 15 min to cross-link histones and DNA, and then glycine was added to stop cross-linking. After washing with PBS three times, the cells were resuspended in SDS lysis buffer with protease inhibitors. After acquiring pelleted nuclei, chromatin was sonicated to shear into 200–500 bp. The supernatants were immunoprecipitated with an H3K27me3 antibody (CST) or a control antibody (anti-IgG) overnight at 4 °C with rotation, which was followed by incubation with protein G agarose for 1 h. Protein G agarose antibody/chromatin complexes were collected, washed, and eluted. Then, cross-links were reversed, and DNA was purified and analysed via real-time PCR. The ChIP-qPCR primer sequences were as follows: *Ngn1* (forward, 5′-tctcccagcccaccaataag-3′; reverse, 5′-tcacagcttgaggtttccat-3′) and *ascl1a* (forward, 5′-ccattgaagccacacgtgaa-3′; reverse, 5′-tgaactgctgctggtttacg-3′).

### Statistical analysis

Statistical significance was performed using GraphPad Prism 5.0 Software. Data were calculated using Student’s *t* tests for comparison of the two groups. One-way analysis of variance (ANOVA) was performed for three or more groups (as in Fig. 5b, c), followed by the LSD post hoc test. Dunnett’s T3 test was used when equal variances were not assumed. All bar graphs were plotted as the mean ± standard error of the mean (SEM), and significance was set at *P* < 0.05.

## Results

### Expression pattern of Sox19b during neuronal development in zebrafish

In this study, zebrafish were selected as an animal model. We first explored the spatiotemporal expression characteristics of the SoxB1 family during zebrafish development by RT-PCR and in situ hybridization. Our results showed that the expression of *SoxB1* mRNA in different stages was as follows: *Sox19b* was highly expressed during the maternal phase, *Sox3* and *Sox19a* were expressed initially in the 1000 cell phase, and *Sox2* was expressed at the beginning of the 30% epiboly phase. All four continued to be expressed at 48 hpf (Fig. 1a). With the development of the zebrafish embryos, the distribution of *Sox19a* and *Sox19b* became gradually concentrated in the neural tube region, especially from the tailbud stage to 48 hpf (Fig. 1b). The maternal expression of Sox19b and the specific neural tube expression, which is similar to Sox2 in mammals, have attracted our attention and emphasize the importance of Sox19b in neural tube development.

### Knockdown of Sox19b caused deficits in the development of neural tubes

**Fig. 2:**
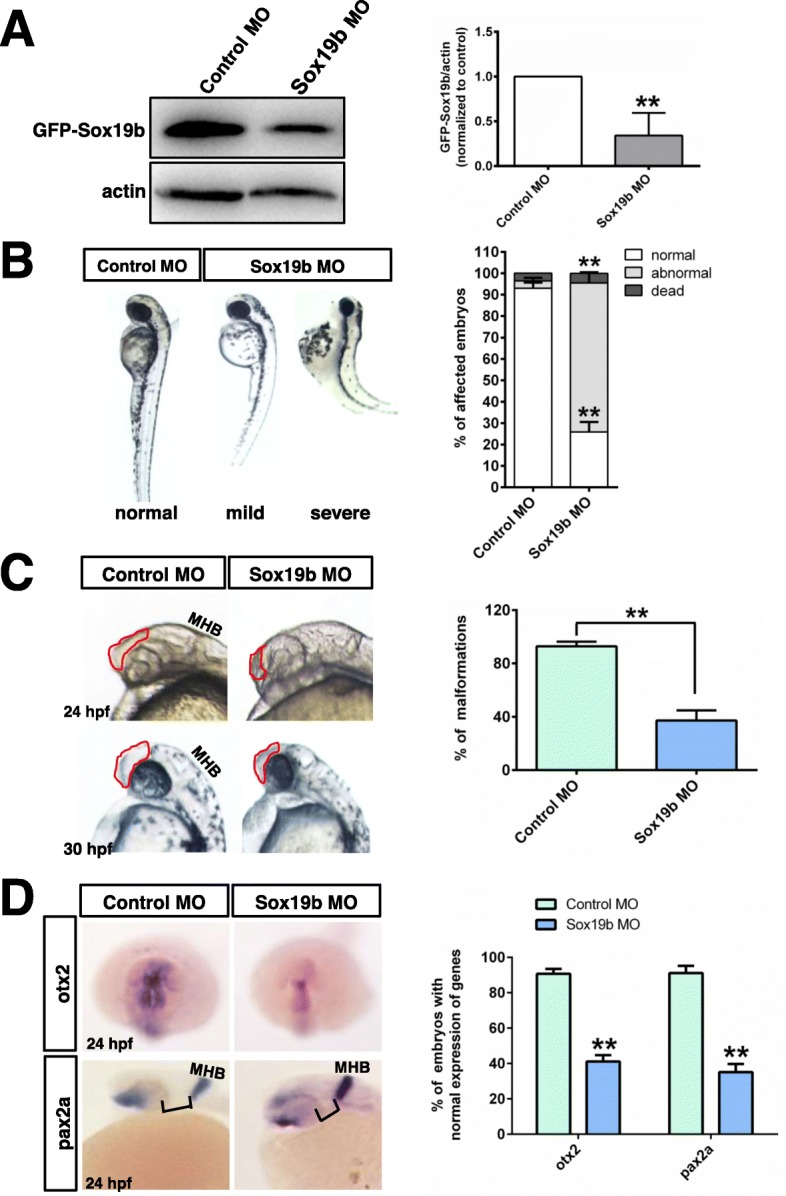
Zebrafish embryos displayed NTDs after knockdown of Sox19b*.* **a** Western blot confirmed that Sox19b MO could interfere *Sox19b* mRNA translation. **b** The neural tube development of zebrafish embryos was abnormal after injection with a Sox19b MO at 48 hpf. There were two main results. The mild phenotype consisted of a smaller head and shorter body axis. The severe phenotype had a partial head area deletion, microphthalmos, shorter body axis, and tail turned up. Bar graphs show the statistical data for the embryo numbers. Data represent the mean of at least three independent experiments ± SD. **P < 0.01 versus control. **c** The zebrafish embryos developed to 24 hpf and 30 hpf, and the embryos injected with Sox19b MO showed that the area of the telencephalon and diencephalon decreased significantly compared with controls. The bar graphs show the percentage of normal embryos in each group. Data represent the mean of at least three independent experiments ± SD. **P < 0.01 versus control. **d** Whole-mount in situ hybridization analysis of neuroectoderm marker expression at 24 hpf. The expression of *otx2* and *pax2a* were decreased in the Sox19b MO group compared with what was observed in the controls. Bar graphs show the percentage of normal embryos in each group. Data represent the mean of at least three independent experiments ± SD. *P < 0.05 versus control

**Fig. 3:**
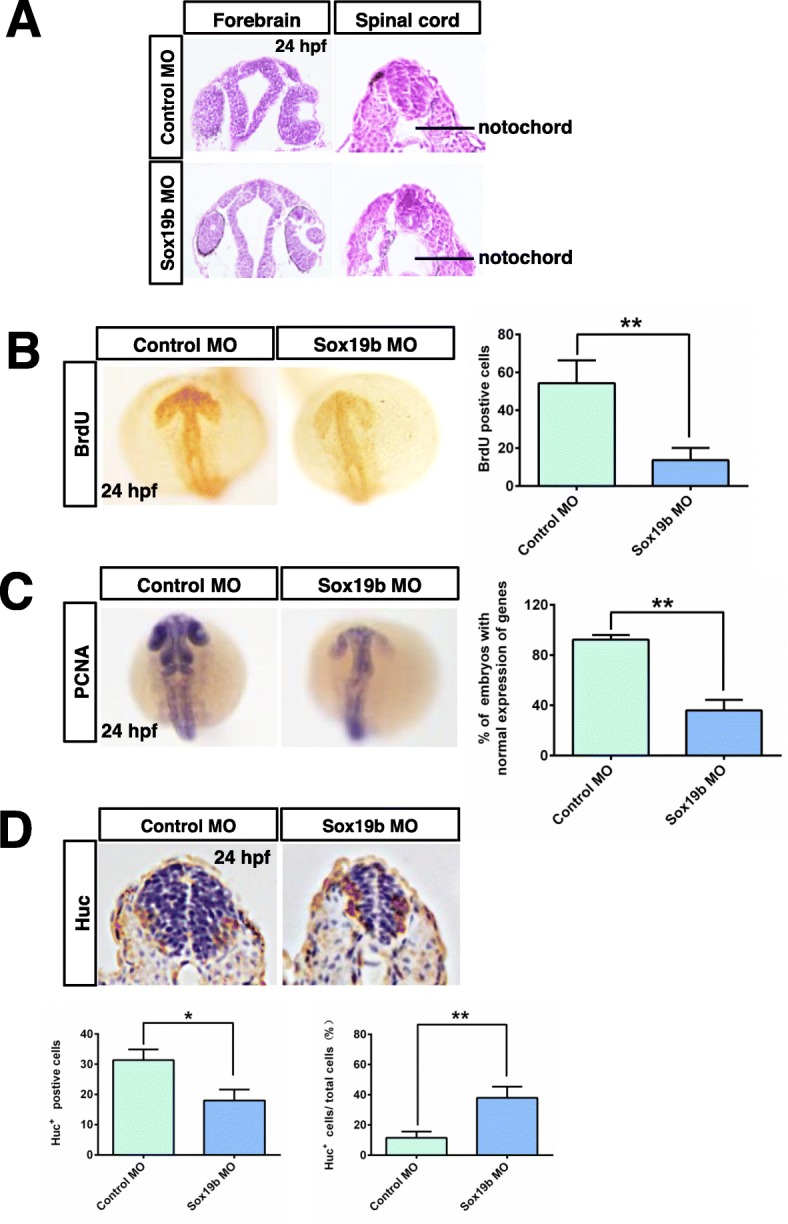
Effect of Sox19b on NSCs in neural tubes. **a** H&E histological sections of the brain and spinal cord of zebrafish embryos at 24 hpf showed that the number of cells and cell layers in the neural tube decreased, and the ventricle expanded in the Sox19b MO-injected embryos compared with controls. **b** BrdU labelling of control MO- and Sox19b MO-injected embryos at 24 hpf. Data represent the mean of at least three independent experiments ± SD. **P < 0.01 versus control. **c** The expression of *PCNA* was detected by whole-mount in situ hybridization, and the expression was used to detect proliferating cells in zebrafish embryos of control MO MO- and Sox19b MO-injected embryos. **d** Immunolabelling of HuC (early neuronal marker) at 24 hpf showed that the expression of HuC was ectopic in Sox19b morphants, and the expression of HuC appeared in some regions that were enriched with NSCs. Data represent the mean of at least three independent experiments ± SD. *P < 0.05 versus control

To verify the role of Sox19b during zebrafish embryonic development, we disrupted its translation using antisense morpholino (MO) oligonucleotides, and the specificity and efficiency of the treatment were assessed. Western blot confirmed that the MO oligonucleotides inhibit the translation of *Sox19b* mRNA (Fig. 2a). We analysed the phenotype and found that zebrafish embryos appeared as follows: the telencephalon and diencephalon were diminished or even lost, the body axis became shorter, and the tail turned up after knockdown of sox19b (Fig. 2b, c). To gain further insights into the molecular mechanism behind this phenotype, we analysed the expression of several markers during neural tube formation. The results showed that the expression levels of both *otx2* (anterior neuroectoderm) and *pax2* (neural ectoderm) were significantly decreased compared with the control group (Fig. 2d). H&E staining of embryo sections at 24 hpf confirmed that the neural tube in the Sox19b MO group became thinner, the lumen was enlarged, and the number of epithelial cells decreased in the forebrain and spinal cord (Fig. 3a).

### Knockdown of Sox19b inhibited the proliferation and induced premature differentiation of NSCs

The proliferation, differentiation, and migration of NSCs are the cytological basis for the normal development of neural tubes. A decrease in the number of epithelial cells means that the proliferation and differentiation of NSCs may be affected. We first detected the effect of Sox19b on the proliferation of NSCs with BrdU incorporation assays. The results showed that the number of BrdU-positive cells was decreased in the Sox19b MO group (Fig. 3b). Moreover, we found that the level of *PCNA*, a well-accepted marker of proliferation, was significantly reduced in the Sox19b MO group (Fig. 3c). We then explored the effect of Sox19b on the differentiation of NSCs. Immunohistochemical staining for HuC showed expression in early neuronal cells, and we found that the absolute quantity of HuC-positive cells decreased. Surprisingly, the proportion of early neuronal cells (HuC^+^ cells/total cells) increased (Fig. 3d), which suggests that the fate of NSCs in the neural tube was abnormal. Taken together, these results suggested that the neural tube defects might be due to the impaired proliferation and differentiation of NSCs after injecting the Sox19b MO.

**Fig. 4:**
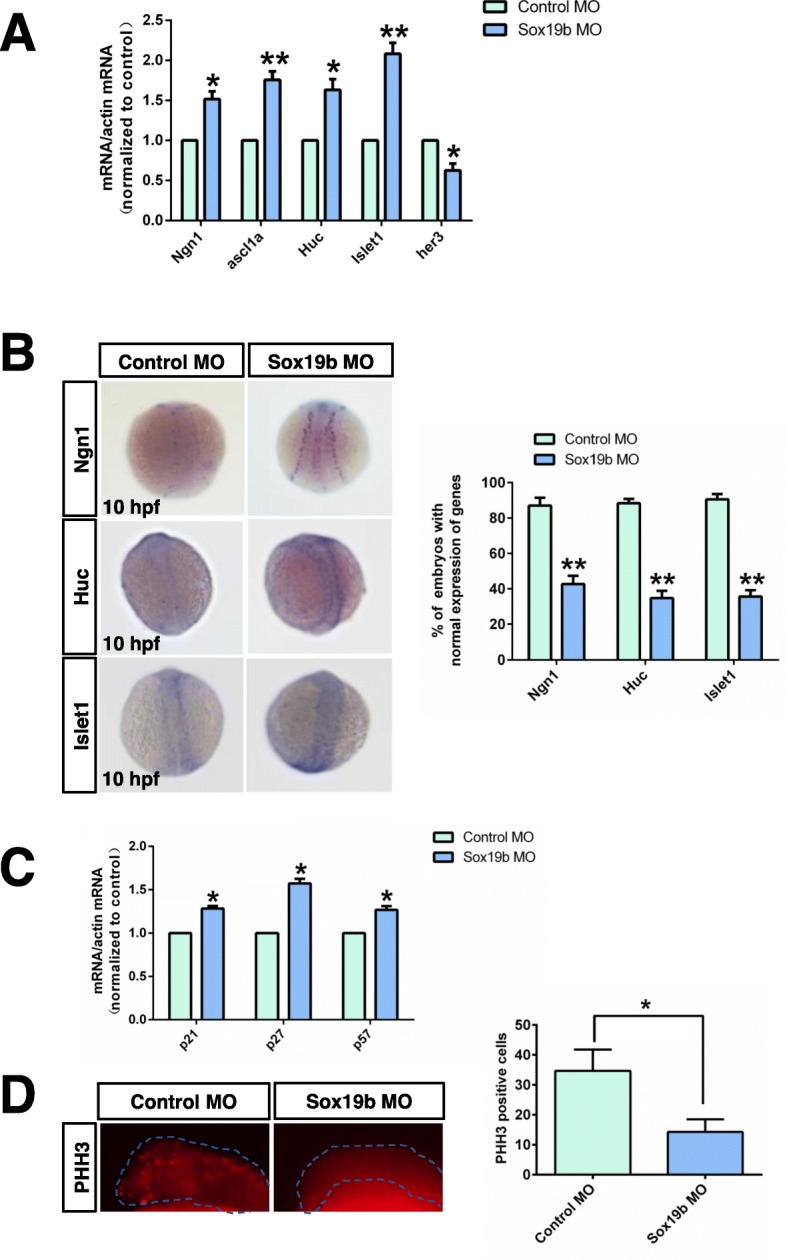
NSCs in embryos injected with *sox19b* MO were prematurely differentiated. **a** Quantitative RT-PCR analysis for *Ngn1*, *ascl1a*, *HuC*, *lslet1*, and *her3*. Compared with the control group, the mRNA levels of *Ngn1*, *ascl1a*, *HuC*, and *lslet1* in Sox19b MO-injection embryos increased significantly, but the mRNA level of *her3* decreased significantly. **b** The expression levels of *Ngn1*, *HuC*, and *lslet1* were detected by whole-mount in situ hybridization. Bar graphs show the percentage of normal embryos in each group. Data represent the mean of at least three independent experiments ± SD. *P < 0.05, **P < 0.01 versus control. **c** Quantitative RT-PCR analysis is shown for *p21*, *p27*, and *p57*. Knockdown of Sox19b caused a significant increase in the mRNA levels of cyclin-dependent kinase inhibitor. Data represent the mean of at least three independent experiments ± SD. *P < 0.05 versus control. **d** Control and Sox19b MO-injected embryos were analysed by immunofluorescence staining of PHH3 (a marker of mitosis, red). Data represent the mean of at least three independent experiments ± SD. *P < 0.05 versus control

Next, we explored the changes in factors related to the fate of NSC differentiation in zebrafish embryos at a critical stage (10–11 hpf). The results showed that the transcription levels of *Ngn1*, *ascl1a*, *HuC*, and *Islet1* were increased, while the transcription level of *Her3* was decreased (Fig. 4a, b). Cyclin-dependent kinase (CDK) inhibitors are important for differentiation. We found that, compared with the control group, the expression of the CDK inhibitors *p21*, *p27*, and *p57* was increased after injecting of Sox19b MO (Fig. 4c). We used a phospho-histone H3 (p-H3) antibody to label the mitotic cells and found that the number of p-H3^+^ NSCs in the Sox19b MO group was significantly smaller than what was seen in the control group (Fig. 4d). These results showed that NSCs were prematurely differentiated, and the number of undifferentiated NSCs was reduced.

### Knockdown of Sox19b inhibited the expression of EZH2 and reduced the trimethylation of histone H3

**Fig. 5:**
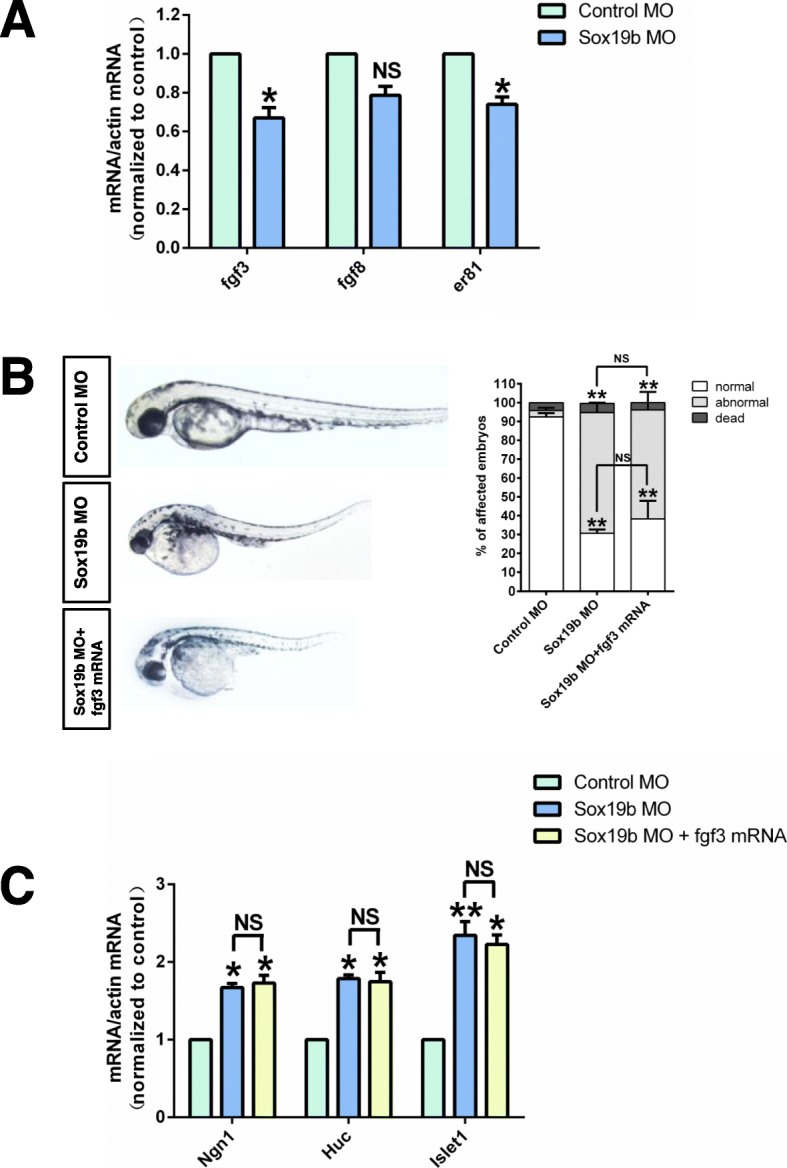
The FGF pathway did not play the most important role in the Sox19b regulation of NSCs. **a** Quantitative RT-PCR analysis is shown for *fgf3*, *fgf8*, and *er81*. Compared with the controls, the mRNA levels of *fgf3* and *er81* in Sox19b MO-injected embryos decreased significantly. Data represent the mean of at least three independent experiments ± SD. *P < 0.05 versus control. **b** Compared with embryos injected with Sox19b MO, the NTD appearance in zebrafish embryos was not rescued by the addition of *fgf3* mRNA. **c** Quantitative RT-PCR analysis for *Ngn1*, *Huc*, and *Islet1*. *Fgf3* mRNA could not change the increase in *Ngn1*, *Huc*, or *lslet1* induced by Sox19b MO treatment. Data represent the mean of at least three independent experiments ± SD. *P < 0.05, **P < 0.01 versus control; ns, no significant difference

In zebrafish, the regulation of NSCs involves the coordination of multiple signalling pathways, such as FGF, Wnt, and BMP. Among them, FGF is mainly involved in the regulation of NSCs from the end of gastrulation. Our results showed that there was a decrease in the activity of the FGF signalling pathway after knocking down Sox19b, but injection with *fgf3* mRNA could not rescue the most obvious phenotype, which was telencephalic loss and premature differentiation of NSCs (Fig. 5a–c). These results suggest that there may be another mechanism by which Sox19b regulates NSCs.

**Fig. 6:**
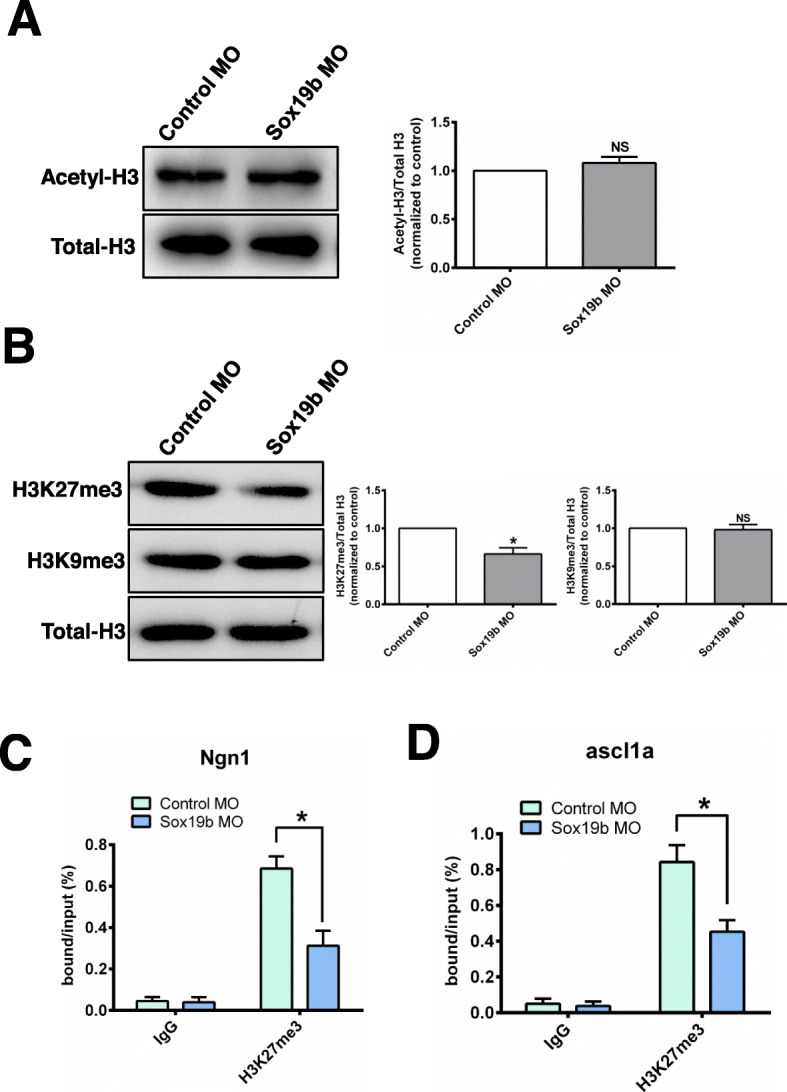
Sox19b regulated NSCs through an epigenetic mechanism. **a** Western blot analysis of acetyl-H3 protein levels in control and Sox19b MO-injected embryos. Sox19b had no effect on the acetyl-H3 levels. **b** Western blot analysis of H3K27me3 and H3K9me3 levels in control and Sox19b MO-injected embryos. The H3K27me3 level decreased after knocking down *sox19b*, but the level of H3K9me3 remained unchanged. Data represent the mean of at least three independent experiments ± SD. *P < 0.05 versus control. **c**, **d** Control and Sox19b MO-injected embryos were immunoprecipitated with anti-H3K27me3 antibody, and the isolated DNA was analysed by using gene-specific ChIP primers. Rabbit IgG was used as a negative control. DNA from each ChIP sample was normalized by the corresponding input sample. Compared with the controls, the level of H3K27me3 in the *Ngn1* and *ascl1a* promoter regions decreased significantly in Sox19b MO-injected embryos. Data represent the mean of at least three independent experiments ± SD. *P < 0.05 versus control

**Fig. 7:**
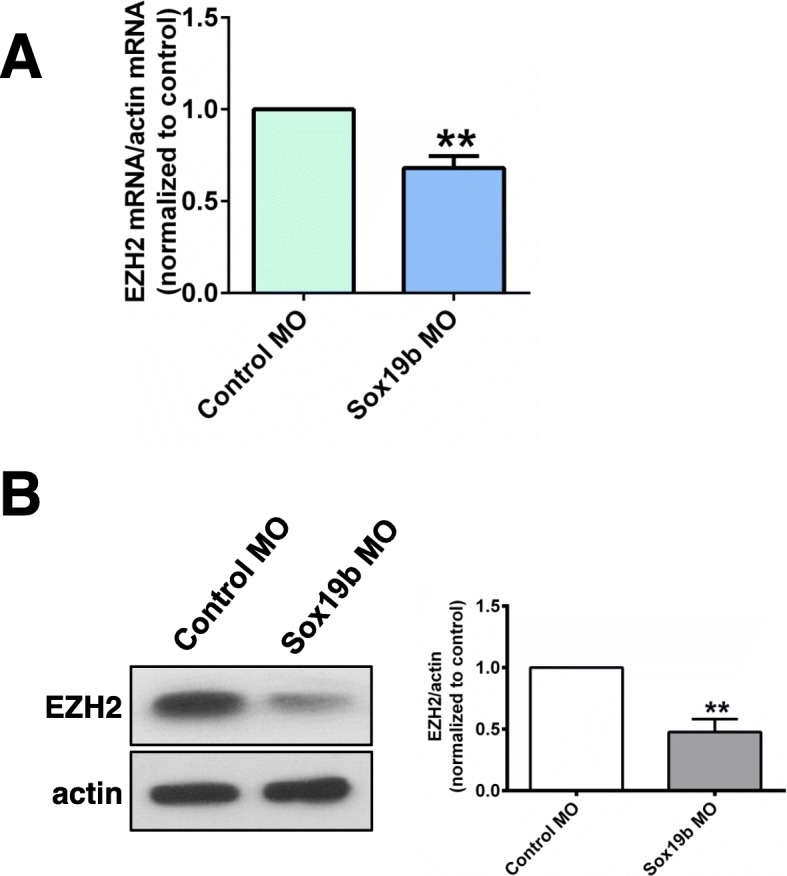
Sox19b regulated NSCs through EZH2. **a** Quantitative RT-PCR analysis is shown for *EZH2*. Compared with the controls, the mRNA level of *EZH2* in Sox19b MO-injected embryos decreased significantly. **b** Western blot analysis of EZH2 protein levels in control and Sox19b MO-injected embryos. The protein level of EZH2 decreased after knocking down of Sox19b. Data represent the mean of at least three independent experiments ± SD. **P < 0.01 versus control

Histone modifications play vital roles in the regulation of gene transcription and embryonic development. It is well known that histone lysine acetylation can significantly promote gene expression, while histone lysine methylation has more complex effects on gene expression (38–40). We first detected the level of histone acetylation and found that the histone acetylation levels in the Sox19b MO group did not change significantly (Fig. 6a). We further found that the level of H3K27me3 in the Sox19b MO group was significantly decreased, but the change of H3K9me3 was not significant (Fig. 6b). The ChIP-qPCR results showed that the levels of H3K27me3 at the promoters of *Ngn1* and *ascl1a* were significantly decreased after injection with the Sox19b MO (Fig. 6c, d). It is known that histone methylation levels are controlled by histone methyltransferase (HMT). The level of H3K27me3 is regulated by the H3K27me3 methylase EZH2. We found that the mRNA and protein levels of EZH2 in the Sox19b MO group were significantly decreased compared with what was observed in the control group (Fig. 7a, b). Overall, we concluded that Sox19b may regulate the differentiation of NSCs through EZH2-mediated regulation of H3K27me3 levels.

**Fig. 8:**
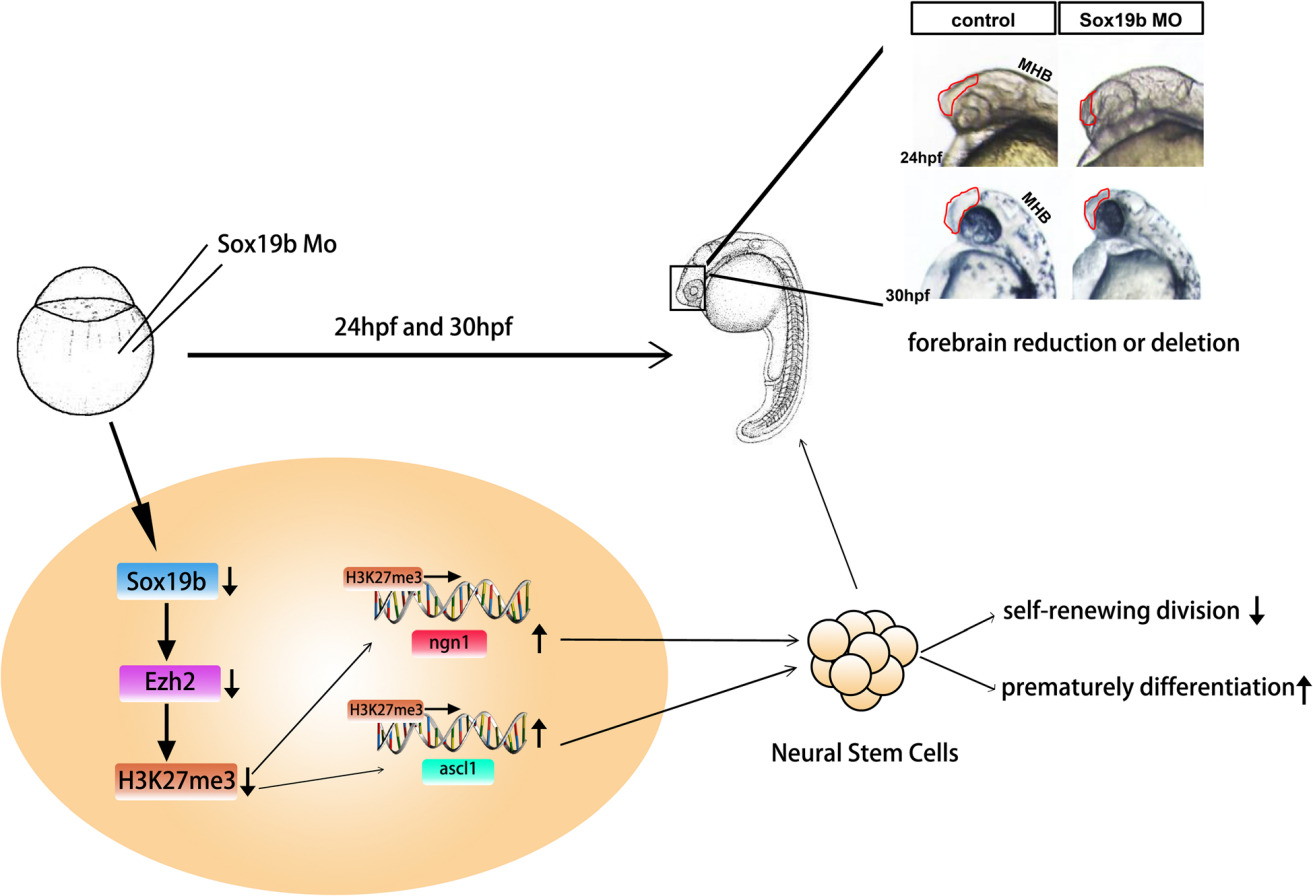
Schematic representation of Sox19b-regulated neural differentiation of NSCs in zebrafish neural tube development

Taken together, we first discovered the role of Sox19b in NTDs (Fig. 8), established the regulatory network of transcription factors and epigenetic factors to NSCs, and provided new clues and theoretical guidance for the clinical treatment of NTDs.

## Discussion

NTDs are among the most common and serious birth defects in humans. Failure of the embryonic process to generate neural tube closure can lead to serious neurological consequences or even lethality [3, 4]. The generation, proliferation, differentiation, and migration of NSCs and the resulting morphogenesis are the cytological basis for neural tube development, and abnormalities in this process lead to neural tube defects [9]. Studies uncovering the molecular regulation of NSCs are of major importance for the prevention and treatment of NTDs. In this study, we used zebrafish as an animal model. We found that loss of the SoxB1 family transcription factor Sox19b resulted in deformities that were similar to neural tube defects in zebrafish embryos. Furthermore, decreased proliferation and increased premature differentiation of NSCs were found in the zebrafish embryo with knocked down Sox19b. This suggests that Sox19b may participate in the development of neural tubes by regulating the transition from proliferation to differentiation and the fate of NSCs. Finally, our results showed that Sox19b regulated the trimethylation of histone H3K27 through EZH2. After inhibition of Sox19b, decreased H3K27me3 levels altered the chromatin state of *Ngn1* and *ascl1a* and reduced their transcription, thereby affecting the differentiation of NSCs.

The SoxB1 family plays the most important role in the proliferation and differentiation of NSCs [16], and its expression is significantly reduced in patients with NTDs and animal models [17–19]. The SoxB1 family in zebrafish includes Sox1a, Sox1b, Sox2, Sox3, Sox19a, and Sox19b [22]. Early studies on zebrafish SoxB1 have focused on Sox2 and Sox3. Sox2 regulates the development of the retina and the lens. Inhibition of Sox2 results in anophthalmos and affects the development of the hypothalamus [24]. Sox3 is mainly involved in the process of neural induction. Overexpression of Sox3 results in the formation of an extra neural plate [25]. However, it has also been reported that zebrafish embryos had no obvious central nervous system (CNS) dysplasia phenotype after inhibiting the expression of Sox2 and Sox3 separately [23]. The above studies show that there are still many controversies in the existing research and the role of the SoxB1 family in zebrafish neural tube development.

Sox19a and Sox19b are unique to bony fish [22]. Sox19a and Sox19b have been continually expressed in the region of the presumptive CNS and appear to be the earliest molecular markers of the CNS [22]. Compared with Sox19a, Sox19b is maternally expressed; its transcription starts at gastrulation, and it is expressed in the early stages of the presumptive neuroectoderm. Later, Sox19b is restricted to the presumptive CNS, including the forebrain, midbrain, hindbrain, and spinal cord [22, 26]. Our results also confirm these expression results. Previous studies have shown that overexpression of Sox19a and Sox19b could repress the expression of organizer genes, and only Sox19b could effectively rescue the effects of a Sox3 dominant-negative construct [27]. In this study, we used MO oligonucleotides to inhibit the expression of Sox19a and Sox19b. The results showed that after the injection of a Sox19b MO, embryos exhibited an obvious neural tube defect phenotype, but there was no obvious abnormality in the embryo injected with a Sox19a MO (data not shown). These results confirmed the specificity of Sox19b function. Previous studies have shown that Sox19b functions as an essential CNS anteroposterior patterning determinant by regulating organizer activity [26]. Sox19b participates in the regulation of neurogenesis in the spinal cord following an injury [28, 29]. In this study, we showed that the embryos injected with a Sox19b MO exhibited a neural tube defect phenotype: the telencephalon and diencephalon were diminished or even missing, and the body was shortened. Furthermore, the expression levels of *otx2* (anterior neuroectoderm) and *pax2a* (neural ectoderm) in the Sox19b MO group were also decreased. The morphological analysis found that the number of cells in the neural tube of the Sox19b MO-injected embryos was decreased, and the chamber was enlarged.

The proliferation, differentiation, and migration of NSCs are the cytological basis for the normal development of neural tubes. A decrease in the number of epithelial cells means that the proliferation and differentiation of NSCs may be affected. First, we examined the effect of Sox19b reduction on the proliferation of NSCs. We found that BrdU-positive cells were decreased and that PCNA was also significantly reduced in the Sox19b MO group. Subsequently, the differentiation of NSCs was also confirmed to be abnormal. The results showed that although the absolute quantity of HuC-positive cells decreased, the ratio of neuronal cells (HuC + cells/total cells) increased. At the time of the formation of the neural plate (10 hpf), the transcription levels of *Ngn1*, *ascl1a*, *HuC*, and *Islet1* were increased, but the transcription level of *her3* was decreased. These results showed that loss of Sox19b resulted in the premature differentiation of NSCs. Meanwhile, the expression levels of the CDK inhibitors *p21*, *p27*, and *p57* were increased, which further suggested that inhibition of Sox19b led to the premature exit of NSCs from the cell cycle. Our data suggested that Sox19b played an important role in the fate determination of NSCs.

In mammals, Sox2 promotes the proliferation of NSCs and maintains the number of NSCs by increasing the transcription of Notch1, RBP-J, and Hes5 in the Notch signalling pathway. Sox3 binds directly to the fgf3 promoter region and regulate its transcription [30, 31]. We detected multiple signalling pathways affecting the proliferation and differentiation of NSCs in zebrafish, such as FGF, Wnt, and BMP. We found that the loss of Sox19b affected the FGF signalling pathway, and the expression levels of *fgf3* and *er81* were significantly decreased. However, injection of *fgf3* mRNA could not rescue the NTD phenotype or the premature differentiation of NSCs.

Increasing evidence has shown that transcription factors and epigenetic modifications work together in regulating gene transcription [32, 33]. Our team conducted a series of studies confirming that covalent modifications of histones are involved in the regulation of NSC neuronal differentiation [34–36]. Covalent modification of histones mainly includes acetylation, methylation, phosphorylation, and ubiquitination of histone H3 and H4 [37]. In this study, we found that inhibition of Sox19b did not affect the acetylation of histone H3, but it did alter the trimethylation of histone H3, mainly the trimethylation of lysine site 27. Removal of this repressive chromatin modification results in marked upregulation of gene expression, the consequence of which is a shift in the balance between self-renewal and differentiation towards differentiation.

## Conclusions

Based on these results, we conclude that Sox19b plays a crucial role in neural tube development and regulates the differentiation fate of NSCs. We first found that Sox19b maintained the level of H3K27me3 through EZH2, ensuring that NSCs would not differentiate prematurely. We have established a regulatory network of transcription factors and epigenetic factors in the fate of NSC differentiation. From the perspective of disease treatment, this study provides a good basis for further revealing the role of the SoxB1 family in the development of NTDs and provides new ideas and new clues for the clinical treatment of NTDs.

## Data Availability

The datasets generated and/or analysed during the current study are included within the article and are available from the corresponding author on reasonable request.

## Abbreviations

NTDs: Neural tube defects
SoxB1: SRY-related HMG box B1
CNS: Central nervous system
NSCs: Neural stem cells
MO: Morpholino
CDK: Cyclin-dependent kinase
